# Cigarette smoke attenuates phagocytic ability of macrophages through down-regulating Milk fat globule-EGF factor 8 (MFG-E8) expressions

**DOI:** 10.1038/srep42642

**Published:** 2017-02-14

**Authors:** Yueqin Wang, Guangwei Luo, Jie Chen, Rui Jiang, Jianhua Zhu, Na Hu, Wei Huang, Guilian Cheng, Min Jia, Bingtao Su, Nian Zhang, Tianpen Cui

**Affiliations:** 1 Laboratory of Clinical Immunology, Wuhan No. 1 Hospital, Tongji Medical College, Huazhong University of Science and Technology, Wuhan, Hubei, P.R. China; 2 Department of Respiratory Medicine, Wuhan No. 1 Hospital, Tongji Medical College, Huazhong University of Science and Technology, Wuhan, Hubei, P.R. China; 3 Department of Pathophysiology, School of Basic Medicine, Tongji Medical College, Huazhong University of Science and Technology, Wuhan, Hubei, P.R. China

## Abstract

Chronic obstructive pulmonary disease (COPD) is one of the most common inflammatory diseases resulting from habitual smoking. Impaired clearance of apoptotic cell by airway macrophages contributes to lung inflammation. Milk fat globule-EGF factor 8 (MFG-E8), as a link between apoptotic cells and phagocytes, facilitates clearance of apoptotic cells and attenuates inflammation. We sought to investigate altered expression and potential role of MFG-E8 in COPD. In this study, apoptosis was increased and the level of MFG-E8 was decreased while HMGB1 expression was increased in lung tissues of CS-exposed mice. Compared with CS-exposed WT mice, more apoptotic cells were accumulated in lung tissues of CS-exposed MFG-E8 deficiency mice. Exposure of a range of macrophages to cigarette smoke extract (CSE) resulted in decreased MFG-E8 expression. Administration of rmMFG-E8 ameliorated phagocytic ability of RAW264.7 cells and suppressed inflammatory response induced by CS-exposure. 10% CSE stimulation suppressed Rac1 membrane localization in RAW264.7 cells which was restored by administration of rmMFG-E8. MFG-E8 deficiency diminished uptake of apoptotic thymocytes by peritoneal macrophages upon CSE exposure. Overall, the findings in current work provide a novel target for diagnosing and treating COPD.

Chronic obstructive pulmonary disease (COPD) is a major cause of morbidity and mortality among respiratory patients, manifested mainly by destruction of alveolar walls and chronic inflammation of airways^1^. Cigarette smoke are regarded as one of risk factors for the development of COPD^1^. Several mechanisms account for the pathogenesis of COPD, including abnormal inflammatory response, oxidative stress, protease/antiprotease imbalance^2^,^3^. Bax and BAD, pro-apoptotic markers, as well as activated subunits of caspase-3, were detected in patients with emphysema^4^. A large number of apoptotic cells including apoptotic endothelial cells, alveolar epithelial cells and inflammatory cells were observed in lung tissues of COPD patients, which ultimately lead to inflammation and destruction of lung tissues^5^. Therefore, apoptosis is proposed to be a novel mechanism implicated in the pathogenesis of COPD.

In healthy individual, apoptotic cells are promptly and effectively removed by professional phagocytes including macrophages, dendritic cells, a process termed efferocytosis^6^. The process is crucial to immune tolerance, tissue homeostasis and resolution of inflammation^7^,^8^. Efferocytosis is an evolutionarily conserved, dynamic process, which is composed of recognition and engulfment of apoptotic cells^9^. Recognition of “eat-me” signal such as phosphatidylserine (PS) on the surface of apoptotic cells is a vital step of efferocytosis. The step was mediated by a broad set of receptors of macrophages and bridging proteins such as growth arrest-specific gene 6 (Gas6) and milk fat globule-epidermal growth factor 8 (MFG-E8)^9^,^10^. Recognition of apoptotic cells triggers activation of signal events involved in cytoskeletal rearrangement, leading to engulfment of apoptotic cells by phagocytes. Rac1, the Rho GTPases, plays an essential role in efferocytosis through promoting formation of protrusion^11^. If apoptotic cells are not timely cleared, they undergo secondary necrosis leading to the release of alarmins such as high-mobility group box-1(HMGB1) and heat-shock proteins, thus induce inflammatory response and autoimmunity^12^. Evidences demonstrated defective efferocytosis contributed to the development of COPD^13^. It was reported that the ability of alveolar macrophages isolated from COPD patients was impaired in phagocytosing apoptotic epithelial cells^13^. Cigarette smoke exposure inhibited efferocytosis of alveolar macrophages through decreasing Rac1 activation^13^,^14^. Identifying the mechanism responsible for defective efferocytosis in COPD will provide new strategies to maintain the homeostatic efferocytosis of alveolar macrophages and prevent the development of COPD.

Milk fat globule-epidermal growth factor 8 (MFG-E8) is a glycoprotein secreted by various types of cells especially phagocytes such as immature dendritic cells, macrophages and epithelial cells^15^,^16^,^17^. MFG-E8, as a bridging protein between phagocytes and apoptotic cells, enhances efferocytosis and ameliorates inflammation^16^,^18^. Binding of MFG-E8-opsonined apoptotic cells to α_v_β_5_ integrin on the surface of phagocytes triggers Rac1 activation thus facilitates efferocytosis^19^. Altered expressions of MFG-E8 were detected in autoimmune diseases, inflammatory diseases, tumors and age-related diseases^20^. It was reported that administration of recombinant MFG-E8 ameliorated inflammatory tissue damage in several experimental models^20^,^21^,^22^. However, little studies focused on the role of MFG-E8 in the development of COPD. In prior study, we demonstrated that the plasma concentration of MFG-E8 in COPD patients was decreased gradually from the Global Initiative for Chronic Obstructive Lung Disease (GOLD) I to GOLDIV^23^. It has also been noted that the levels of MFG-E8 were negatively related with the amount of smoking^23^. In the current study, we aimed to explore the altered expressions of MFG-E8 in CS-exposed mice or macrophages, the effect of MFG-E8 on phagocytic ability and inflammatory response of macrophages. This may provide a novel target for diagnosing and treating COPD.

## Results

### Cigarette smoke exposure induces accumulation of apoptotic cells *in vivo*

**Figure 1:**
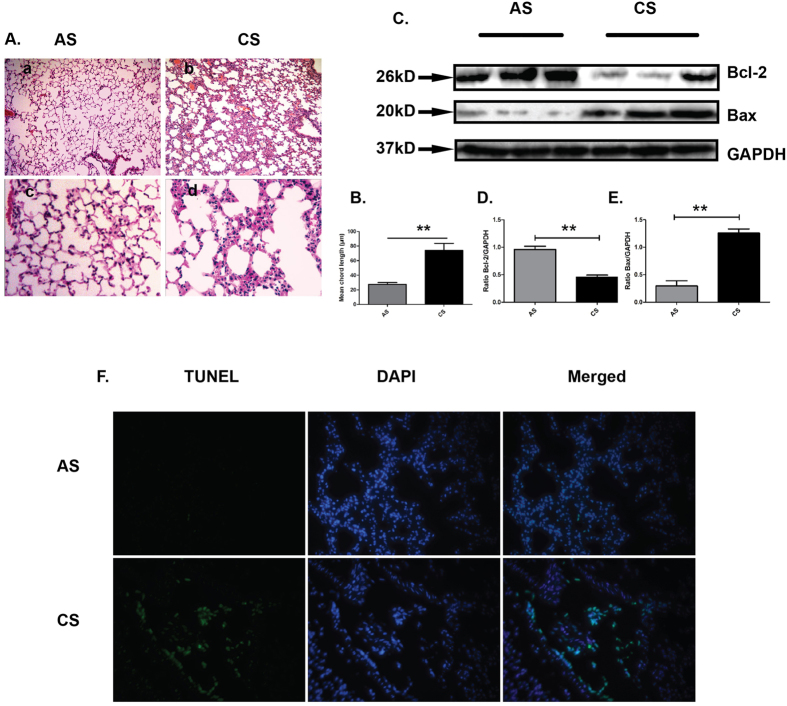
Cigarette smoke exposure induced accumulation of apoptotic cells *in vivo*. (**A**) Morphological changes in lung sections of CS-exposed (n = 10) or room air exposed (n = 10) mice were assessed by H&E staining (a–d). Original magnification: a.b x100, c,d x400. (**B**) The mean of cord length was calculated. Data was mean ± SEM. (**C**) Protein extracted from the lung tissues was subjected to detect the expressions of apoptosis-associated proteins including Bcl-2 and Bax with western blot and GAPDH served as loading control. Cropped blots are shown, the corresponding full-length blots are shown in Figure S1(a–c). Statistic analysis was performed using pooled data from two independent experiments with three independent samples in each group. Data was mean ± SEM. (**D**) Bcl-2; (**E**) Bax. *P < 0.05, **P < 0.01. (**F**) The presence of apoptotic cells in lung tissues was detected with TUNEL staining (green) and counterstained with DAPI(blue), original magnification: x400 **P < 0.01 (AS: room air stimulation CS: cigarette smoke stimulation).

In current study, cigarette smoke exposed mice were used as an animal model for COPD. C57BL/6j mice were divided into CS (exposed to cigarettes smoke, n = 10) and AS (air exposed mice, n = 10) groups. After challenged with cigarette smoke for nine months, the lung sections exhibited apparent pathological changes characterized by airspace enlargement and inflammatory cells infiltration similar to that of COPD patients (Fig. 1A,B).

In attempt to explore whether cigarette smoke exposure induced cellular apoptosis, apoptosis was examined using Western blot and TUNEL aasay. Compared to AS mice, the expression of Bcl-2, an anti-apoptosis marker, was significantly decreased, whereas Bax, a pro-apoptosis marker, was significantly increased in lung sections of CS-exposed mice (Fig. 1C–E). In addition, increased numbers of TUNEL-positive cells were accumulated in lung sections of CS-exposed mice as compared to AS mice (Fig. 1F). Above results indicated that occurrence of apoptosis was increased in lung of CS-exposed mice.

### Cigarette smoke exposure down-regulates MFG-E8 expressions and up-regulates HMGB1 expressions *in vivo*

**Figure 2:**
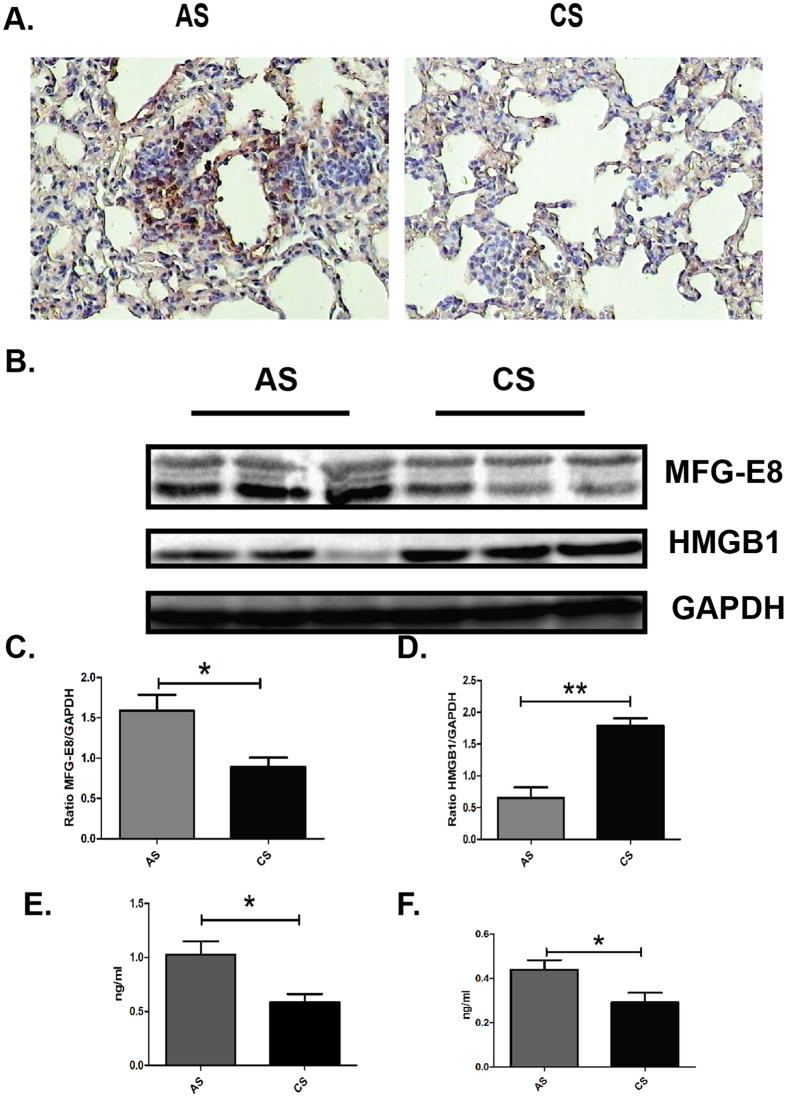
Cigarette smoke exposure down-regulated the levels of MFG-E8 and up-regulated the expressions of HMGB1 *in vivo*. (**A**) The levels of MFG-E8 in lung sections were assessed by immunohistochemistry assay, Original magnification: x200. (**B**) Protein levels of MFG-E8 and HMGB1 in lung tissues were detected with western blot, GAPDH was regarded as internal control. Cropped blots are shown, the corresponding full-length blots are shown in Figure S2(a–c). Densitometric analysis was performed using pooled data from two independent experiments with three independent samples in each group, Data was expressed as mean ± SEM. (**C**) MFG-E8; (**D**) HMGB1. *P < 0.05, **P < 0.01. The levels of MFG-E8 in (**E**) bronchoalveolar lavage fluid (BALF) and (**F**) plasma were measured by ELISA. Date was expressed as mean ± SEM *P < 0.05.

Accumulation of apoptotic cells resulted from imbalance of apoptosis and clearance of apoptotic cells in the development of COPD^13^. MFG-E8, forming a link between apoptotic cells and phagocytes, enhances clearance of apoptotic cells and suppresses inflammatory responses^16^. In previous study, we demonstrated that the plasma level of MFG-E8 was decreased in COPD patients and negatively related with smoking amount^23^. In the current study, we investigated the alterations of MFG-E8 expression in CS-exposed mice. MFG-E8 expression was markedly reduced in lung tissues of CS-exposed mice compared to controls (Fig. 2A–C). In addition, the concentration of MFG-E8 in BALF of CS-exposed mice was much lower than in AS mice (0.58 ± 0.079 *vs* 1.027 ± 0.12 ng/ml, Fig. 2E). Consistent with the previous results, it was found that the plasma MFG-E8 level in CS-exposed mice was lower compared with AS controls (0.29 ± 0.43 *vs* 0.44 ± 0.41 ng/ml, Fig. 2F). HMGB1, competing with MFG-E8 through engaging α_V_β_3_-integrin, negatively regulates clearance of apoptotic cells^17^. In this study, it was shown that HMGB1 expression was increased in lung tissues of CS-exposed mice compared with AS mice (Fig. 2B,D). Above results indicated that CS-exposure down-regulated MFG-E8 expressions.

### MFG-E8 deficiency enhances accumulation of apoptotic cells induced by CS exposure

**Figure 3:**
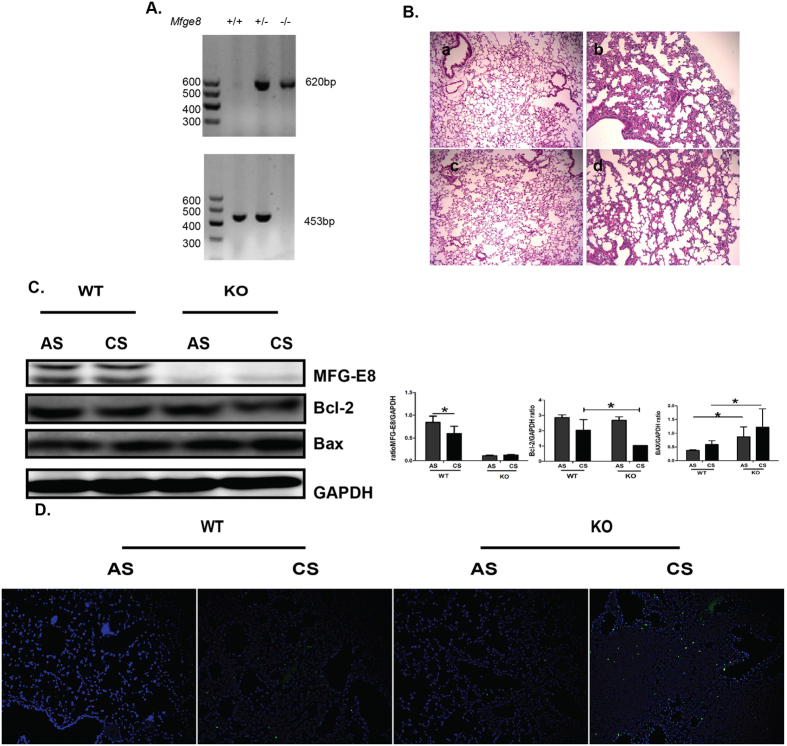
More apoptotic cells were accumulated in the lung tissue of MFG-E8 KO mice than WT mice. (**A**)The mutant of MFG-E8 was assessed by PCR. (**B**) Morphological changes in lung section were assessed by H&E staining. a:room air exposed WT mice; b:CS-exposed WT mice; c:room air exposed MFG-E8 KO mice; d:CS-exposed MFG-E8 KO mice. Original magnification: x100. (**C**) The expressions of MFG-E8 and apoptosis-related markers including Bcl-2 and Bax were measured with western blot. Cropped blots are shown, the corresponding full-length blots are shown in Figure S3(a–d). Statistic analysis was performed using pooled data from two independent experiments with three independent samples in each group. Data was mean ± SEM. *P < 0.05, **P < 0.01. (**D**). The presence of apoptotic cells in lung tissues was detected with TUNEL staining (green) and counterstained with DAPI(blue), original magnification: x200 (AS: room air stimulation CS: cigarette smoke stimulation).

To further assess the effect of MFG-E8 on accumulation of apoptotic cells in lung tissues of CS-exposed mice, we constructed the MFG-E8 KO mice and investigated the pathological changes and apoptosis in MFG-E8 KO mice. The mutant of MFG-E8 was identified by PCR and western blot (Fig. 3A,C). The pathological changes including airspace enlargement and inflammatory cells infiltration were exhibited in lung tissues of CS-exposed MFG-E8 KO mice which is similar to that observed in lung tissues of CS-exposed WT mice (Fig. 3B). Compared with CS-exposed WT mice, Bcl-2 expression was dramatically decreased and Bax expression was increased in lung tissues of CS-exposed MFG-E8 KO mice (Fig. 3C). More importantly, more apoptotic cells were presented in lung tissues of MFG-E8 KO mice (Fig. 3D). Above results indicated that MFG-E8 deficiency contributed to the accumulation of apoptotic cells in lung of CS-exposed mice.

### Stimulation with CSE down-regulates MFG-E8 expressions in macrophages

**Figure 4:**
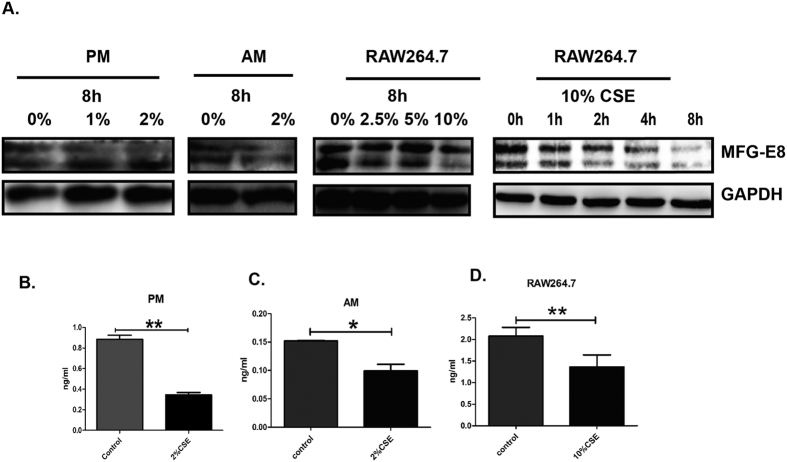
Stimulation with CSE down-regulated the expressions of MFG-E8 in a range of macrophages. A range of macrophages including peritoneal macrophages (PM), alveolar macrophages(AM) and RAW264.7 were stimulated with indicated concentrations of CSE and harvested at indicated time points. The experiments were repeated for three times. (**A**) The expressions of MFG-E8 were assessed with western blot and GAPDH was used as loading control. Cropped blots are shown, the corresponding full-length blots are shown in Figure S4 (a,b). The culture supernatant was collected to measure the concentrations of MFG-E8. (**B**–**D**) (**B**) PM, (**C**) AM, (**D**) RAW264.7. Data was expressed as mean ± SEM (n = 3). *P < 0.05, **P < 0.01. (CSE: cigarette smoke extract).

The effect of cigarette smoke exposure on MFG-E8 levels was further characterized in a range of macrophages. Peritoneal and alveolar macrophages isolated from WT mice were treated with 1% or 2% CSE for 8 hours and it was shown that MFG-E8 protein expression was significantly reduced (Fig. 4A). At the same time, the level of MFG-E8 was down-regulated in culture supernatants from 2% CSE-stimulated primary macrophages, as compared to control group (Fig. 4B,C). RAW264.7 cell, a mouse macrophage cell line, was treated with different concentrations of CSE for 8 hours. The results indicated that the expression of MFG-E8 was remarkably decreased in the 10% CSE exposed RAW264.7 cell lysates as compared to other groups. Meanwhile, the concentrations of MFG-E8 in culture supernatants of 10% CSE exposed RAW264.7 cells were also lower than in control groups (1.30 ± 0.38 *vs* 2.10 ± 0.28 ng/ml, Fig. 4A,D). The study also demonstrated that stimulation with CSE down-regulated MFG-E8 expressions in RAW264.7 cells in a time-dependent manner (Fig. 4A).

### Administration of rmMFG-E8 ameliorated phagocytic ability of RAW264.7 cells and suppressed inflammatory response induced by CS-exposure

**Figure 5:**
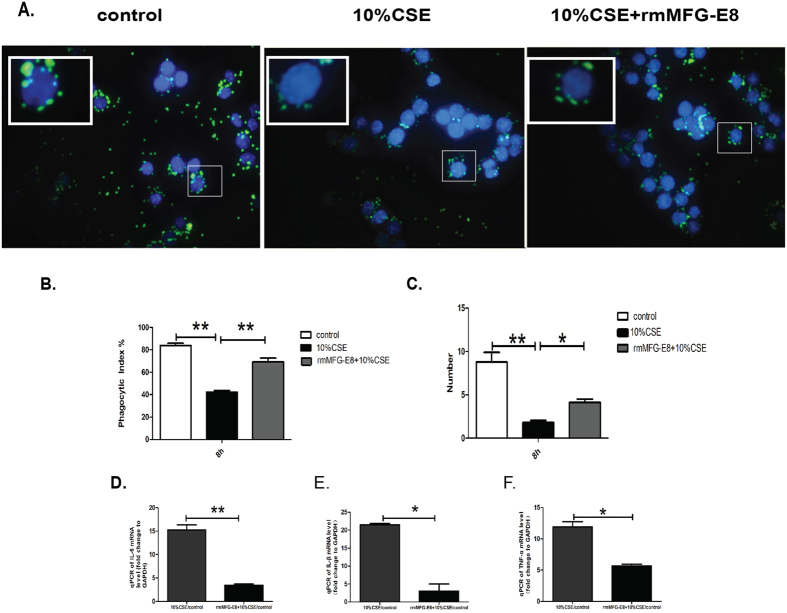
Administration of rmMFG-E8 ameliorates impaired phagocytic ability and inflammatory response induced by 10%CSE in RAW264.7 cells. RAW264.7 cells were stimulated with medium only (control), 10% CSE or rmMFG-E8 (500 ng/ml) + 10% CSE for 8 hours, then fed with FluoSpheres® Carboxylate-Modified Microspheres in the 1:1000 ratio for 90 minutes, counterstained with DAPI(blue). The experiments were repeated for three times. (**A**) Impaired phagocytosis of RAW264.7 induced by 10% CSE stimulation was ameliorated by treatment with rmMFG-E8. Similar results were observed in three independent experiments and representative images at x1000 magnifications were shown. A selected field was enlarged to exhibit the phagocytic ability of RAW264.7. The phagocytic index (**B**) and the numbers of beads engulfed by RAW264.7 cells (**C**) were calculated in ten fields of fluorescence microscope images. Data was expressed as mean ± SEM (n = 3) *P < 0.05, **P < 0.01. The expressions of pro-inflammatory cytokines including (**D**) IL-6, (**E**) IL-1β, and (**F**) TNF-α were detected by real time PCR, data were normalized with GAPDH. Data was expressed as mean ± SEM (n = 3). *P < 0.05, **P < 0.01.

In order to verify whether MFG-E8 plays a role in ameliorating phagocytic ability of CSE-exposed RAW264.7 cells, the cells were pretreated with rmMFG-E8(500 ng/ml) followed by 10% CSE challenge for 8 hours and then fed with FluoSpheres® Carboxylate-Modified Microspheres. CSE-stimulated RAW264.7 cells exhibited decreased ability to phagocytose microspheres (Fig. 5A,B). In addition, fewer microspheres were engulfed by CSE-stimulated RAW264.7 cells compared to controls (Fig. 5A–C). As expected, the ability of CSE-stimulated RAW264.7 cells to phagocytose microspheres was recovered by compensating MFG-E8 in CSE stimulated RAW264.7 cells (Fig. 5A–C).

The expressions of pro-inflammatory cytokines including TNF-α, IL-1β and IL-6 were detected by real time PCR. The levels of TNF-α, IL-1β and IL-6 were significantly elevated in CSE stimulated RAW264.7 cells as compared to controls. Of note, the administration of rmMFG-E8 showed a trend suppressing the elevated expressions of TNF-α, IL-1β and IL-6 in 10% CSE stimulated RAW264.7 cells (Fig. 5D–F). Above results indicated that MFG-E8 played an important role in ameliorating phagocytic ability and suppressing inflammatory responses of macrophages under CS-exposure.

### Administration of rmMFG-E8 restores Rac1 membrane recruitment in CSE stimulated RAW264.7 cells

**Figure 6:**
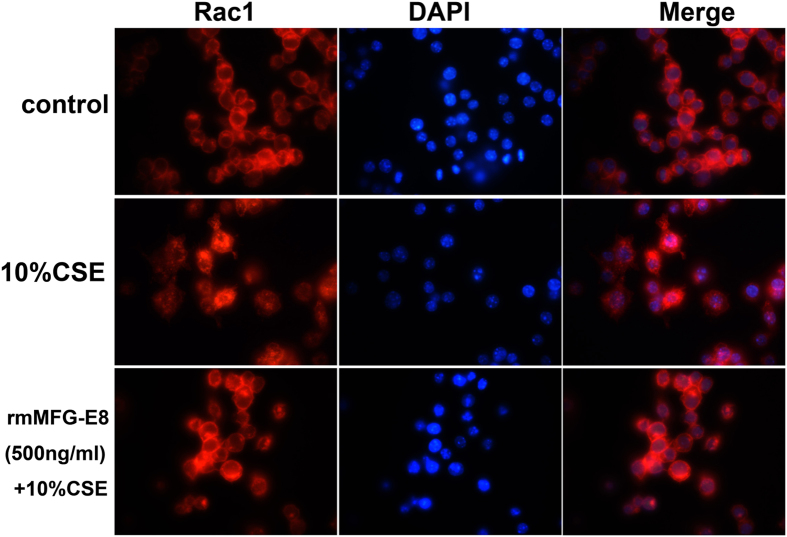
Administration of rmMFG-E8 restored Rac1 membrane recruitment in 10% CSE stimulated RAW264.7 cells. Subcellullar location of Rac1 (red) was assessed by immunocytochemistry. 10%CSE stimulation inhibited Rac1 membrane location, rmMFG-E8(500 ng/ml) partly provoked the translocation of Rac1 from cytosol to the membrane. Representative fluorescence images at x1000 original magnifications from three independent experiments were shown.

It was reported that MFG-E8 was implicated in regulating phagocytosis through its interaction with Crk-DOCK180-Rac1 pathway^19^. Rac1 aggregates at the cell membrane which promotes formation of protrusion, thus plays an essential role in phagocytosis^24^. In the present study, 10% CSE stimulation suppressed Rac1 membrane localization in RAW264.7 cells. Administration of rmMFG-E8 enhanced the translocation of Rac1 from the cytosol to the membrane in CSE-stimulated RAW264.7 cells, implicating the essential role of MFG-E8 in phagocytosis of macrophages (Fig. 6).

### MFG-E8 deficiency diminishes uptake of apoptotic thymocytes by peritoneal macrophages upon CSE exposure

**Figure 7:**
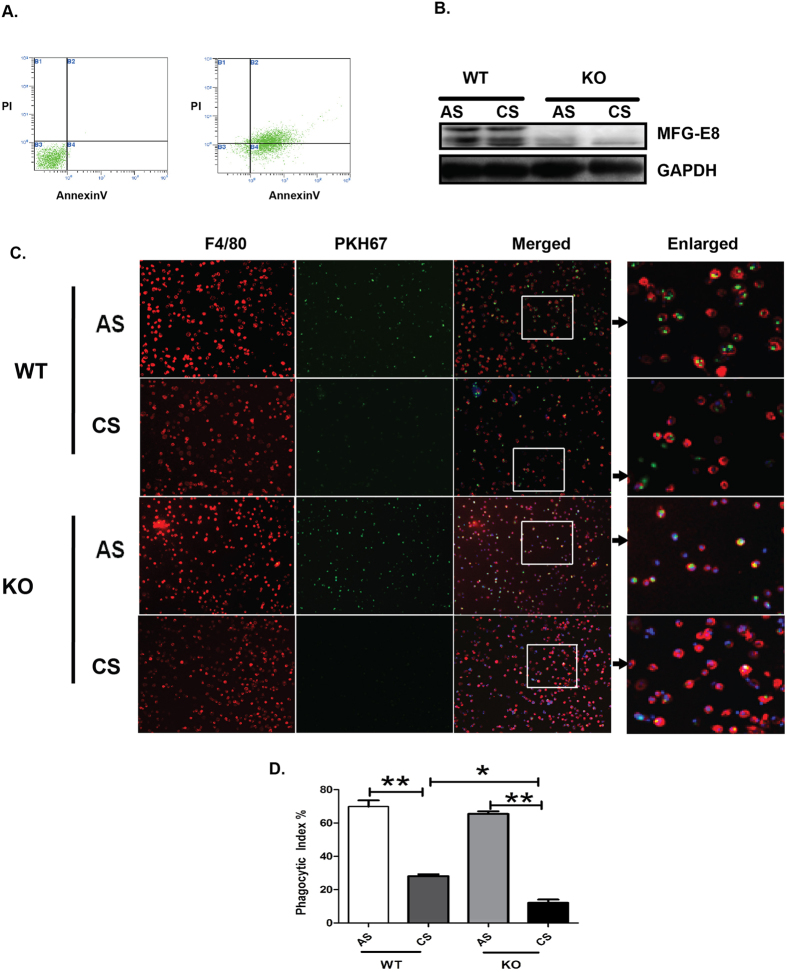
MFG-E8 deficiency diminishes uptake of apoptotic thymocytes by peritoneal macrophages upon CSE exposure. (**A**) The percentage of apoptotic thymocytes was assessed by staining with FITC-labelled AnnexinV/PI. Representative images were shown from three independent experiments. (**B**) The expressions of MFG-E8 were detected with western blot. Representative blot were shown from three independent experiments, the corresponding full-length blots are shown in Figure S5(a,b). (**C**) WT or MFG-E8 KO peritoneal macrophages were treated with or without 2%CSE for 8 hours, followed by incubation with fluorescently labeled apoptotic thymocytes. Representative fluorescence images at x100 original magnifications from three independent experiments were shown. A selected field was enlarged to present the phagocytic ability of PM. (**D**) The phagocytic index was calculated in ten fields of fluorescence microscope images. Data was expressed as mean ± SEM (n = 3)*P < 0.05, **P < 0.01.

To further confirm the effect of MFG-E8 on engulfment of apoptotic cells, peritoneal macrophages isolated from WT or MFG-E8 KO mice were treated with or without 2% CSE for 8 hours, followed by incubation with fluorescently labeled apoptotic thymocytes. Phagocytosis index was quantified as the percentage of macrophages engulfing at least one labeled apoptotic cell. Decreased expression of MFG-E8 was detected in WT peritoneal macrophages treated with 2% CSE (Fig. 7B). In addition, treatment with CSE profoundly inhibited engulfment of apoptotic cells by WT peritoneal macrophages (Fig. 7C). Under the normal culture condition, no difference in engulfment of apoptotic thymocytes was detected between WT peritoneal macrophages and MFG-E8-null peritoneal macrophages, however, under 2% CSE exposure, the engulfment of apoptotic cells by MFG-E8-null macrophages was dramatically reduced compared with WT peritoneal macrophages (Fig. 7C). Taken together, these results demonstrated that MFG-E8 deficiency contributed to decreased ability of macrophages to engulf apoptotic cells.

## Discussion

COPD is characterized by chronic inflammation of airway and destruction of alveolar wall^2^,^25^. Cigarette smoke inhalation is strongly associated with the development of COPD^1^. Cigarette-smoke-derived toxins can cause an increase in oxidative stress, promote inflammatory response and apoptosis^3^. In the current study, chronic cigarette smoke-exposed mice was used as an animal mode for COPD, which presented the same pathological changes including airspace enlargement and inflammatory cells infiltration with COPD patients. In lung tissues of CS-exposed mice, we noticed that Bcl-2 expression, anti-apoptosis protein, was decreased and Bax, pro-apoptosis protein, was increased, more apoptotic cells were observed. Both increased occurrence of apoptosis and impaired phagocytosis of alveolar macrophages led to accumulation of apoptotic cells in lung of cigarette smoke-exposed mice^26^. Treatments to reduce accumulation of apoptotic cells and ameliorate the phagocytosis of macrophages could be potential therapies for COPD.

MFG-E8 facilitates the clearance of apoptotic cells and suppresses inflammatory response, thus participates in maintaining balance in multiple physiological systems^16^. Evidences demonstrated that aberrant MFG-E8 expressions were observed in numberous diseases and associated with the pathogenesis of these diseases. Recent studies showed that the levels of MFG-E8 in patients with SLE-type autoimmune disease or tumors were elevated, whereas, the expressions of MFG-E8 in inflammatory diseases such as inflammatory bowel disease, sepsis were reported to be lower^20^,^27^,^28^,^29^. Substantial studies have focused on the mechanisms of MFG-E8 involved in development of acute lung injury. The levels of MFG-E8 were markedly reduced in asthma patients and MFG-E8 deficiency resulted in airway hyperresponsiveness in murine model of asthma^30^. In LPS-induced acute lung injury models, MFG-E8 attenuated neutrophil infiltration via modulation of CXCR2^31^. In addition, MFG-E8 decreased the severity of tissue fibrosis through binding and targeting the collagen for uptake by macrophages^32^. To date, little studies have investigated the effect of MFG-E8 in the development of chronic pulmonary injury. In previous study, we demonstrated for first time that plasma levels of MFG-E8 in COPD patients were decreased and negatively associated with the amount of smoking, which implies MFG-E8 may play an important role in COPD^23^. In this study, we further explored the altered expressions and effect of MFG-E8 in COPD. Decreased MFG-E8 expressions and elevated HMGB1expressions were observed in lung tissues of CS-exposed mice. More apoptotic cells were observed in lung tissues of MFG-E8 deficiency mice. It was well characterized that increased levels of HMGB1 were observed in inflammatory conditions, and associated with the development of tissue injury^33^. MFG-E8 has been shown to reduce the severity of inflammation^34^. In addition, HMGB1 was reported to compete with MFG-E8 for binding to α_v_β_3_, and diminish the ability of macrophages to engulf apoptotic cells^17^. The aforementioned study indicated decreased expressions of MFG-E8 might be associated with the accumulation of apoptotic cells in lung sections of CS-exposed mice.

To reveal whether MFG-E8 is involved in phagocytosis and inflammatory response of macrophages under CS-exposure, we established an *in vitro* model by exposing macrophages to CSE. Consistant with the effect of CS-exposure on MFG-E8 expressions *in vivo*, CSE exposure decreased the expressions of MFG-E8 in both primary macrophages and macrophage cell line. The effect of MFG-E8 on inflammatory response has been extensively investigated, MFG-E8 inhibits release of pro-inflammatory cytokines such as TNF-α, IL-1β and IL-6 by suppress MAPK, NF-κB, ERK1/2 activation, thus plays a vital role in anti-inflammation^16^,^34^,^35^. As demonstrated in the study, CSE exposure induced the transcription of pro-inflammatory cytokines including IL-6, TNF-α and IL-1β, which were down-regulated by administration of rmMFG-E8.

In the current study, CSE exposure impaired the ability of RAW264.7 cells to engulf Microspheres, Administration of rmMFG-E8 rescued phagocytosis of RAW264.7 cells. Furthermore, we also assessed the effect of MFG-E8 on the capability of macrophages to uptake apoptotic cells, less apoptotic cells were engulfed by MFG-E8-null peritoneal macrophages compared with WT peritoneal macrophages under CS-exposure. Cytoskeletal reorganization is a prominent event involved in phagocytosis. The Rho family of GTPases, composed of Rac1, Cdc42 and RhoA, has been acknowledged as regulators involved in cytoskeletal reorganization, Rac1, aggregating on the plasma membrane, forms membrane protrusions thus favors phagocytosis^36^,^37^. As shown in a previous study, MFG-E8 interacting wih αvβ5 integrin and DOCK180 activated Rac1 to participant in phagocytosis of macrophages^19^. In this study, CSE exposure inhibited location of Rac1 in plasma membrane, however, administration of rmMFG-E8 restored Rac1 membrane recruitment, which indicated that MFG-E8 rescued ability of CSE-exposed phagocytes through restoring Rac1 membrane location.

To sum up, the current study demonstrated for the first time that MFG-E8 deficiency enhanced the accumulation of apoptotic cells in lung of CS-exposed mice. In addition, the current study delineated a novel role of MFG-E8 in phagocytosis and inflammatory response of macrophages under CS-exposure. The study indicated that MFG-E8 might be a potential target to diagnose and treat COPD.

## Methods

### Experimental model

Male C57BL/6j Wild-type (WT) mice were purchased from Center for Animal Experiment, Wuhan University. MFG-E8 KO mice were constructed on a C57BL/6j background by deleted exon III to exon IIV genomic fragment generated by Nanjing Biomedical Research Institute of Nanjing University (NBRI, China)^38^. To identify the *Mfge8* gene by PCR, tail clipping and mouse genomic DNA were extracted with Animal tissue DNA Kit (SEMEN, China). Primers for MFG-E8 wild type were 5′-TTGCCAACAGGCTTGATGGATAT-3′ and 5′-GACAGTACGGAA CAGCGAAGGTA-3′. Primers for MFG-E8 knockout were 5′-GTGGGCAAGTGCATCTGAGTAC-3′and 5′-GAGCGATCCTATCTCAAAACCA3′. Mice were divided into CS (exposed to cigarettes smoke, n = 10) and AS (air exposed mice, n = 10) groups.

WT mice or MFG-E8 KO mice in CS group were exposed to the mainstream smoke of five cigarettes (each containing 12 mg of tar and 1.25 mg of nicotine; Hongjinglong brand, Wuhan, China), twice a day, 5 days a week in an inhalation box for 9 months. In AS control group, mice were exposed to room air only. The mice were sacrificed to collect lung tissues and bronchoalveolar lavage fluid (BALF). All animal experimental procedures were approved by the Committee on Animal Research of Tongji Medical College, Huazhong University of Science and Technology and conducted in accordance with the National Institute of Health Guide for the Care and Use of Laboratory Animals.

### Histological examination and immunohistochemistry

For histological examination, paraffin-embedded lung sections (4 μm) were stained with hematoxylin and eosin (H&E). MFG-E8 protein expression was evaluated by immunohistochemistry. Briefly, paraffin-embedded lung sections (4 μm) were incubated with primary MFG-E8 antibody at a 1:100 dilution (MBL, Nagoya, Japan), followed by incubating with biotinylated-anti-mouse IgG (Dako, Glostrup, Denmark), then colored with 3,3-diaminobenzidine and counterstained with haematoxylin.

### TUNEL assay

The presence of apoptotic cells in lung tissues of mice was assessed with terminal transferase deoxyuridine triphosphate nick-end labeling (TUNEL) staining (Roche Diagnostics, Indianapolis, IN) in accordance to instructions of the manufacturer. In brief, paraffin-embedded lung sections (4 μm) were deparaffinized followed by digesting with 20 μg/ml Proteinase K, incubated with TUNEL reaction mixture containing enzyme solution and label solution, counterstained with DAPI (Beyotime, Nanjing, China), subsequently mounted under coverslips. The images were observed and captured using fluorescent microscope.

### Preparation of cigarette smoke extract (CSE)

Commercial cigarettes (Hongjinglong brand, Wuhan, China) were used to prepare CSE. Briefly, cigarette smoke from one cigarette were bubbled through 10 ml DMEM medium in glass bottle and mixed by shaking, followed by adjusting pH to 7.4. The CSE solution was passed through 0.22 μm filter to remove large particles. The solution was defined as 100% CSE. Working concentration was made by diluting the 100% CSE with culture medium.

### Cell culture and CSE treatment

RAW264.7 cells, obtained from American Type Culture Collection (ATCC), were seeded in 6-well plates in Dulbecco’s modified Eagle’s medium (DMEM) containing 10% fetal bovine serum. Twenty-four hours post-seeding, RAW264.7 cells were stimulated with 2.5%, 5%, 10% CSE, and harvested at indicated time points. To evaluate biological effect of MFG-E8, RAW264.7 cells were pre-treated with rmMFG-E8 (500 ng/ml) (R&D systems) for two hours followed by 10% CSE challenge for the subsequent experiments.

### Isolation of peritoneal and alveolar macrophages and treatment with CSE

Peritoneal and alveolar macrophages were collected from WT or MFG-E8 KO mice (6–8 weeks) euthanized by 1% sodium pentobarbital. Peritoneal macrophages were collected by washing peritoneal cavities of mice with 5 ml of RPMI 1640 medium. Alveolar macrophages were collected by consecutively washing with 1 ml PRMI 1640 medium. Cells were isolated by centrifuge respectively and cell pellets were resuspended in RPMI 1640 medium containing 10% fetal bovine serum to seed in 12-well plates. Four hours post-seeding, all non-adherent cells were removed by washing with PBS twice, the adherent cells were identified as macrophages by morphology assessment and anti-F4/80 staining (BD Biosciences, Sydney, Australia). Twenty-four hours post-seeding, the peritoneal and alveolar macrophages collected from WT or MFG-E8 KO mice were exposed to 1%, 2% CSE respectively and harvested at special time points for the subsequent experiments.

### ELISA for MFG-E8 levels

Detection of MFG-E8 in BALF of experimental models and culture supernatant was performed by ELISA with mouse MFG-E8 protein ELISA kits (R&D Systems) in accordance to the maufacturer’s instructions.

### Western blot

Homogenized lung tissues and cells lysed in lysis buffer (50 mM Tris-HCl pH 8.0, 1 mM EDTA, 250 mM NaCl, 1% NP-40 and 0.5% Na-Deoxycholate) supplemented with protease inhibitor cocktail (Roche Diagnostics). Protein concentration was measured using a commercial kit (Beyotime, Nanjing, China). 30 μg of protein was separated on SDS-PAGE gel and transferred to ployvinylidene difluoride (PVDF) membrane and then incubated with primary antibody against MFG-E8 (MBL, Nagoya, Japan), HMGB1 (Abcam, Cambridge, MA), GAPDH (Santa Cruz Biotechnology, Santa Cruz, CA), Bcl-2 (Cell Signaling Technologies, Danvers, MA), Bax (Abcam, Cambridge, MA) respectively, Subsequently incubated with appropriate HRP-labeled secondary antibody (R&D Systems). The intensities of the bands were quantified with Bio-Rad Imaging system (Bio-Rad, USA).

### Phagocytosis of microspheres

RAW264.7 cells were seeded at a density of 4 × 10^5^ per well on coverslips in 6-well plates, twenty-four hours post-seeding, RAW264.7 cells were pre-treated with or without 500 ng/ml rmMFG-E8 ((R&D Systems) for two hours, and then exposed to 10% CSE for 8 hours. After changing culture medium, FluoSpheres® Carboxylate-Modified Microspheres (Invitrigen, USA) were added to RAW264.7 cells at ratios of 1000:1. After co-culture for 90 minutes, unengulfed microspheres were removed by rising with PBS for three times, fixed in 4% paraformaldehyde, counterstained with DAPI to visualize nucleus. The phagocytic index was defined as the percentage of RAW264.7 cells engulfing one or more microspheres.

### Preparation of apoptotic thymocytes

Thymuses were dissected from 6–8 week old male C57BL/6J mice euthanized by 1% sodium pentobarbital. Thymocytes were collected by grinding thymus through a cell strainer (40 um, BD). Thymocytes were incubated with dexamethasone (0.1 uM) for 16 hours in DMEM. The percentage of apoptotic thymocytes was assessed by staining with FITC-labelled AnnexinV/PI according to the manufacturer’ instructions, followed by flow cytometry.

### Phagocytosis of apoptotic thymocytes

4 × 10^5^ peritoneal macrophages collected from WT or MFG-E8 KO mice were seeded on coverslips. Twenty-four hours post-seeding, the cells were treated with 2% CSE for 8 hours. After changing medium, 4 × 10^6^ apoptotic thymocytes labeled with PKH-67 green fluorescent dye were added to the peritoneal macrophages and co-cultured for 2 hours. Cells were fixed and stained with Cy3-labbed antibody against F4/80. The images were visualized and obtained using a fluorescence microscope. Phagocytic index was calculated as the percentage of peritoneal macrophages ingesting at least one apoptotic thymocytes.

### Real time PCR

Transcripts of inflammatory cytokines including IL-6, T NF-α and IL-1β were measured by Real time PCR. Total RNA was isolated from RAW264.7 cells by TRIzol Reagent (Invitrogen, USA) and 1 μg of RNA was reverse transcribed to cDNA with ReverTra Ace qPCR RT kit (TOYOBO, Shanghai, China). Gene expression was quantified using Platinum^®^SYBR^®^Green qPCR SuperMix UDG Kit (Invitrogen, USA) on the Real-Time PCR Detection System (Bio-Rad, USA). All assays were performed in duplicates for three independent experiments. Specifical primers (Sangon Biotech, Shanghai, China) used in this study were listed as followed: mouseTNF-α:(forward,5′-AGACCCTCACACTCAGATCATCTTC-3′; reverse,5′-TTGCTACGACGTGGGCTACA-3′), mouseIL-1β:(forward,5′-TCATTGTGGCTGTGGAGAAG-3′; reverse,5′-AGGCCACAGGTATTTTGTCG-3′), mouseIL-6:(forward,5′-CGCTATGAAGTTCCTCTCTGCAA-3′;reverse,5′-CACCAGCATCAGTCCCAAGA-3), mouseGAPDH:(forward,5′-ACCCAGAAGACTGTGGATGG-3′;reverse,5′-CACATTGGGGTAGGAACAC-3′). Relative expression of target gene was analyzed using established ^ΔΔ^Ct threshold method (ΔCT = CT_Target_ − CT_GAPDH_, ΔΔCT = ΔCT_Test_ − ΔCT_Control_).

### Immunocytochemistry

RAW264.7 cells grown on coverslips were pretreated with or without rmMFG-E8 (500 ng/ml) (R&D systems) for 2 hours and then stimulated with or without 10% CSE for 8 hours. Cells were fixed with 4% paraformaldehyde, incubated with primary antibody against Rac1 (BD Biosciences, Sydney, Australia) at 1:100. Subsequently, the coverslips were stained with Cy3-labeled rabbit anti-mouse IgG, then counterstained with DAPI. The images were visualized and obtained using a fluorescence microscope.

### Statistical analysis

Statistical analyses were performed using GraphPad Prism Software (La Jolla, CA, USA). Unpaired t-test was applied to comparisons between two groups and one-way ANOVA was used for comparisons between more than two groups. Data were expressed as mean ± SEM. A *p* value of <0.05 was regarded significant.

## Additional Information

**How to cite this article**: Wang, Y. *et al*. Cigarette smoke attenuates phagocytic ability of macrophages through down-regulating Milk fat globule-EGF factor 8 (MFG-E8) expressions. *Sci. Rep.* **7**, 42642; doi: 10.1038/srep42642 (2017).

**Publisher's note:** Springer Nature remains neutral with regard to jurisdictional claims in published maps and institutional affiliations.

## Supplementary Material

Supplementary Information
