# Supplementary material for: Cigarette smoke attenuates phagocytic ability of macrophages through down-regulating Milk fat globule-EGF factor 8 (MFG-E8) expressions
Source: Sci Rep. 2017 Feb 14;7:42642. doi: 10.1038/srep42642 (PMC5307389; doi:10.1038/srep42642)
Supplement: Supplementary Information [file srep42642-s1.pdf]

Cigarette smoke attenuates phagocytic ability of macrophages through down-regulating Milk fat globule-EGF factor 8 (MFG-E8) expressions

Yueqin Wang*[1], Guangwei Luo*[2], Jie Chen[1], Rui Jiang[1], Jianhua Zhu[1], Na Hu[1], Wei Huang[3],Guilian Cheng[1], Min Jia[1], Bingtao Su[1], Nian Zhang[2], Tianpen Cui[1]

[1] Laboratory of Clinical Immunology, Wuhan No.1 Hospital, Tongji Medical College, Huazhong University of Science and Technology, Wuhan, Hubei, P.R. China

[2] Department of Respiratory Medicine, Wuhan No.1 Hospital, Tongji Medical College, Huazhong University of Science and Technology, Wuhan, Hubei, P.R. China.

[3]Department of Pathophysiology, School of Basic Medicine, Tongji Medical College, Huazhong University of Science and Technology, Wuhan, Hubei, P.R. China.

*These authors contributed equally to this work.

Correspondence and requests for materials should be addressed to T.C.(E-mail: tianpencui@126.com)

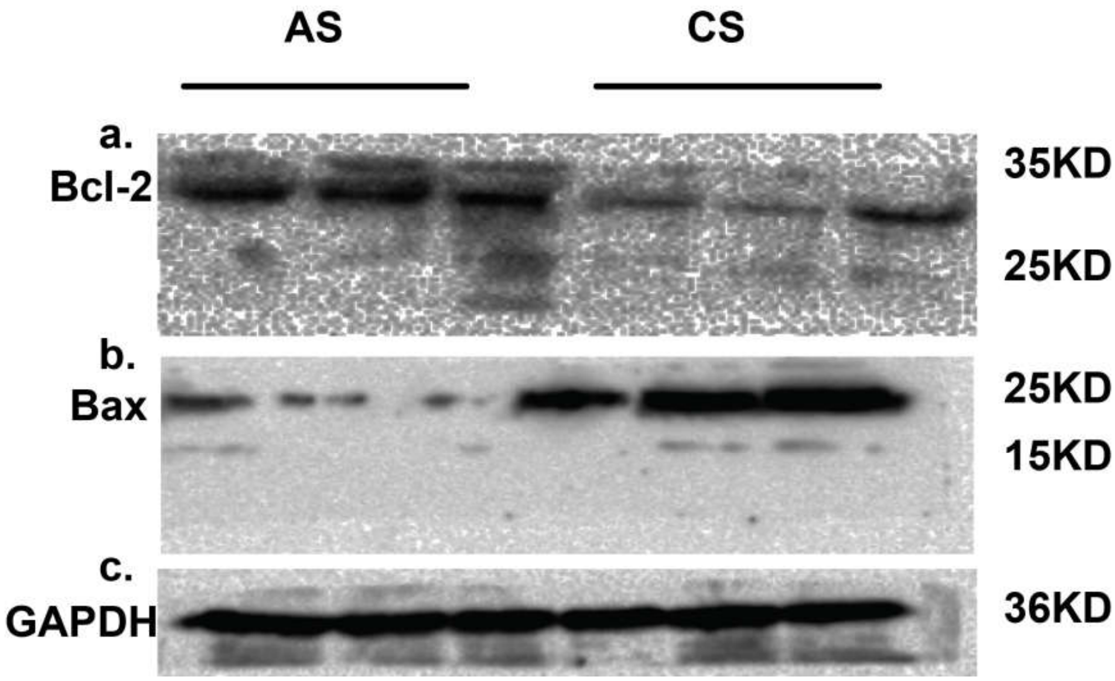

Figure S1 related to Figure 1C

Male C57BL/6j wild-type (WT) mice were exposed to the cigarette smoke or room air for 9 months, the protein extracted from the lung tissues was subjected to detected the expressions of apoptosis-associated proteins including Bcl-2(a), Bax(b) with western blot, GAPDH(c) was served as the loading control. Based on the molecular weight of proteins, the membrane was cut into a few parts and incubated indicated antibody.

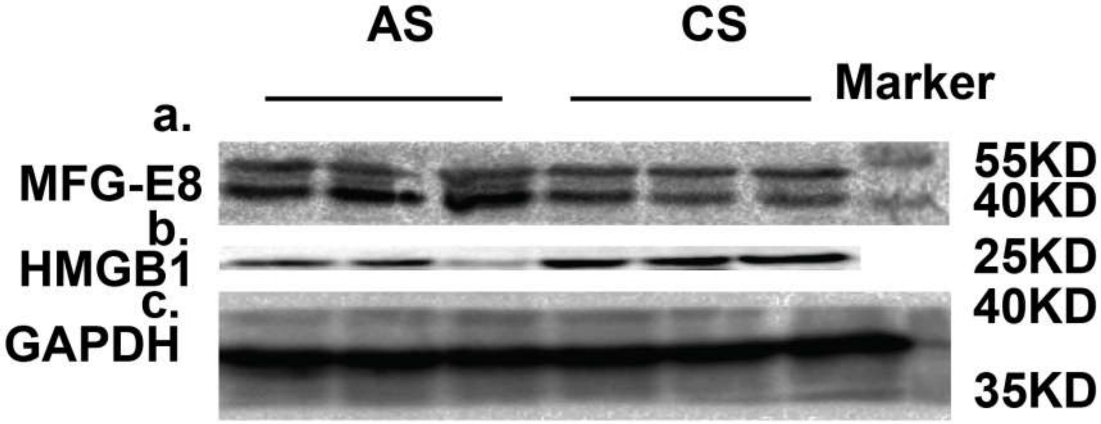

Figure S2 related to Figure 2B

Male C57BL/6j wild-type (WT) mice were exposed to the cigarette smoke or room air for 9 months, western bolt was used to evaluate the levels of MFG-E8(a) and HMGB1(b) in the lung tissues of mice. GAPDH(c) was as regarded the internal control. Based on the molecular weight of proteins, the membrane was cut into a few parts and incubated indicated antibody.

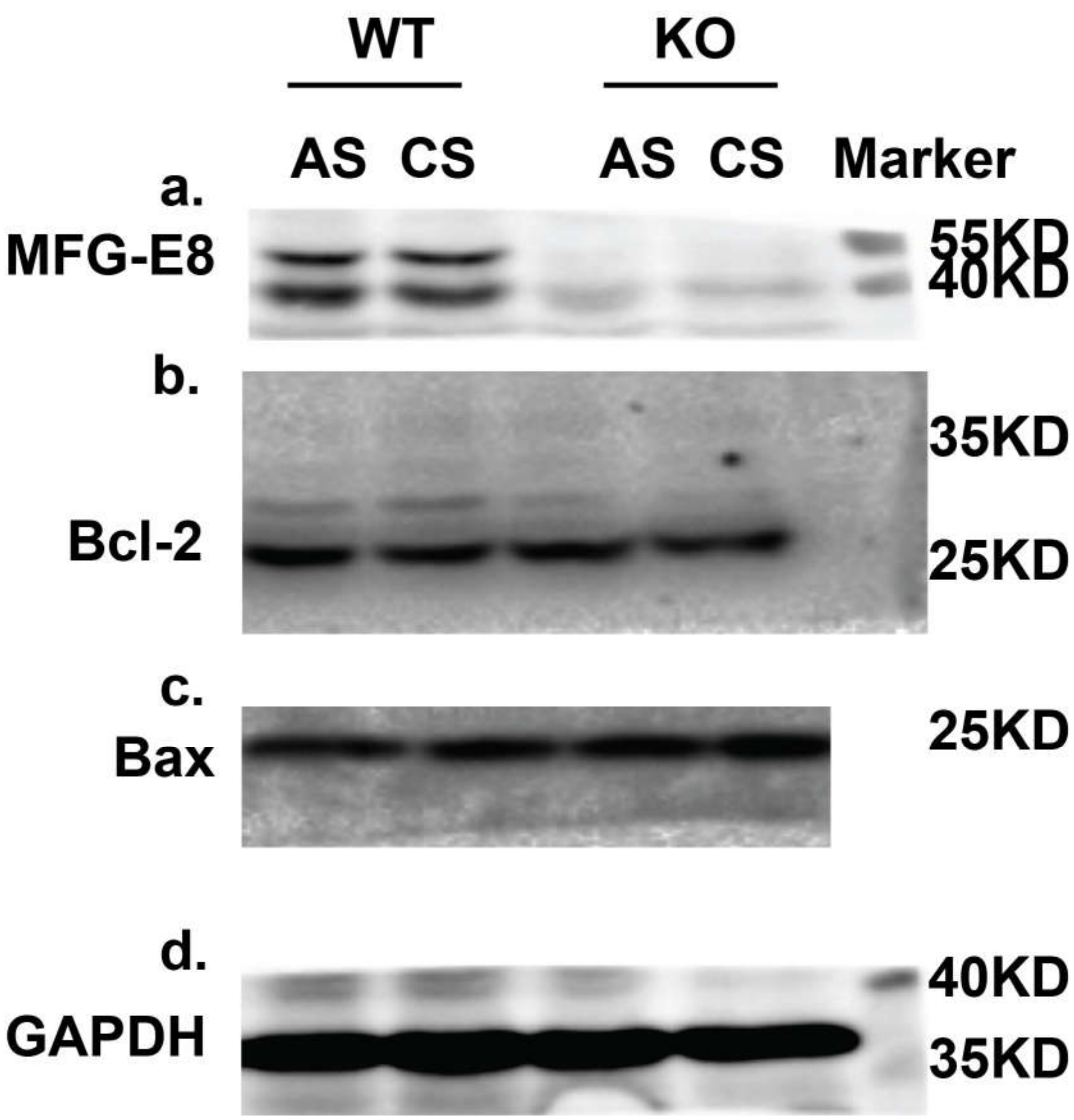

Figure S3 related to Figure 3C

WT mice or MFG-E8 KO mice were exposed to the cigarette smoke or room air for 9 months. The expressions of MFG-E8(a) and apoptosis-related proteins including Bcl-2(b), Bax(c) in the lung tissues of mice were assessed with western blot. GAPDH(d) was regarded as loading control. Based on the molecular weight of proteins, the membrane was cut into a few parts and incubated indicated antibody.

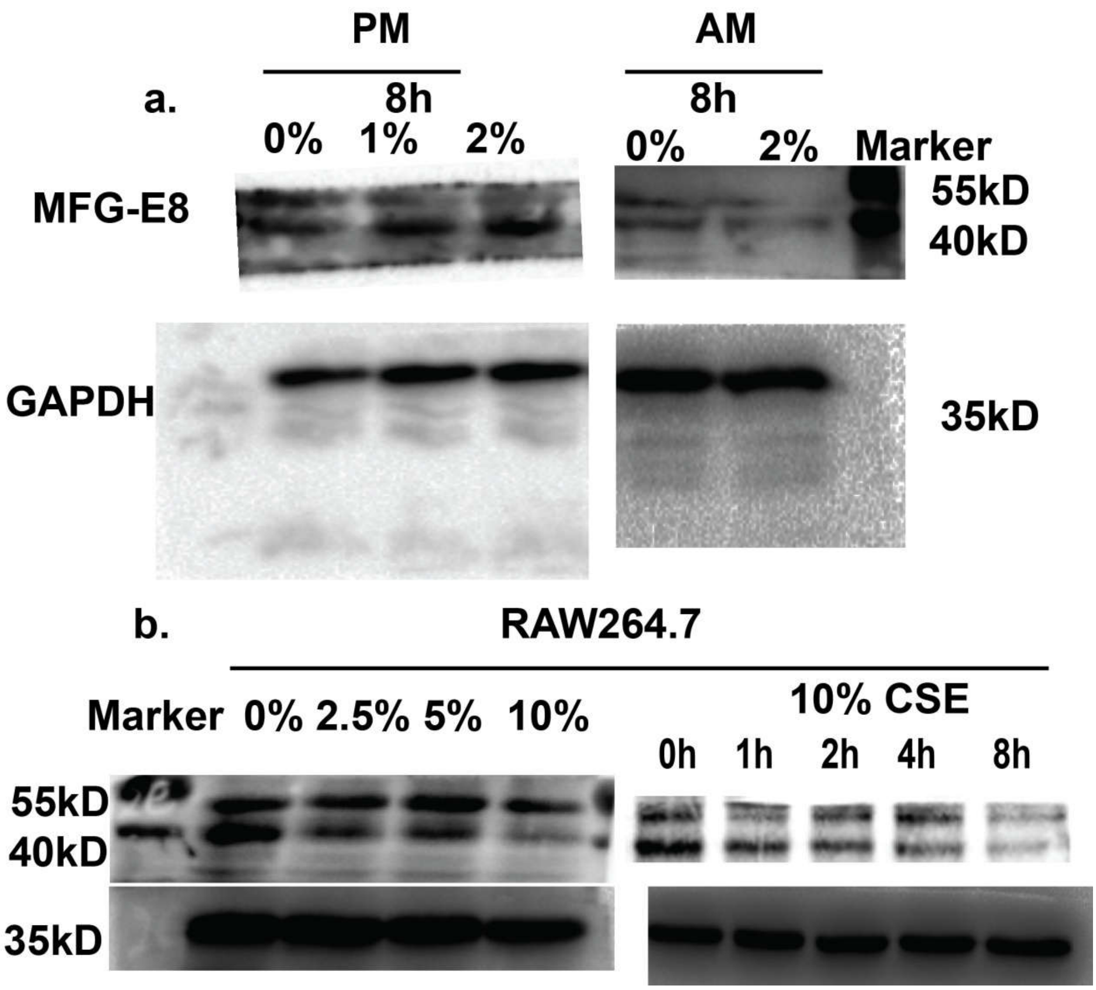

Figure S4 related to Figure 4A

peritoneal macrophages(PM), alveolar macrophages(AM) and RAW264.7 were stimulated with indicated concentrations of CSE and harvested at indicated time points. The expressions of MFG-E8 in PM(a), AM(a), and RAW264.7 cells(b) were detected with western blot. GAPDH was regarded as the internal control. Based on the molecular weight of proteins, the membrane was cut into a few parts and incubated indicated antibody.

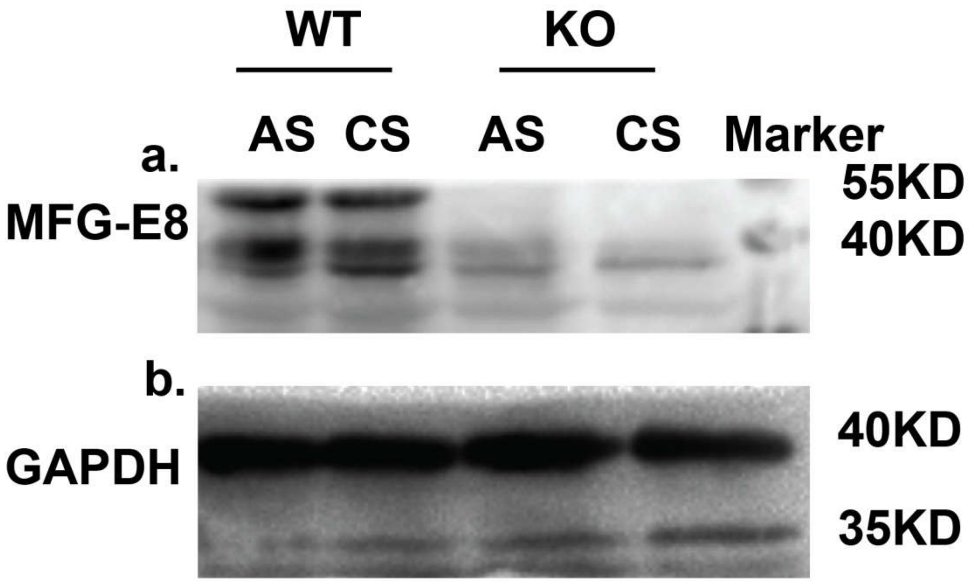

Figure S5 related to Figure 7B

peritoneal macrophages isolated from WT or MFG-E8 KO mice were exposed to 2%CSE for 8hours and western blot was used to detected the expressions of MFG-E8(a). GAPDH (b)was as the loading control. Based on the molecular weight of proteins, the membrane was cut into a few parts and incubated indicated antibody.
